# Cognitive and behavioral risk factors for child physical abuse among Chinese children: a multiple-informant study

**DOI:** 10.1186/s13034-016-0124-5

**Published:** 2016-10-06

**Authors:** Naixue Cui, Jianghong Liu

**Affiliations:** University of Pennsylvania, Room 426 Fagin Hall, 418 Curie Blvd, Philadelphia, PA 19104 USA

## Abstract

**Background:**

It has been well established that child physical abuse is a risk factor for cognitive deficits and behavioral problems. However, the possible link between cognitive deficits and behavioral problems placing children at a higher risk of physical abuse has been overlooked. Using a prospective design, the present study aims to examine whether previously measured cognition indicated by intelligence quotient (IQ), including performance IQ (PIQ) and verbal IQ (VIQ), and behavioral problems reported by multiple informants (i.e. mothers, teachers, and children) predict later child physical abuse (which may include minor and severe forms of abuse inflicted separately by mothers and fathers) in Chinese children.

**Methods:**

A school-based survey was conducted to collect data from 265 Chinese children (52.8 % boys, mean age 13.71 ± 0.60 years) in the Wave II of China Jintan Cohort study. When they were in the last year of elementary school, children completed the Chinese version of the Wechsler intelligence scale for children-revised that measured VIQ and PIQ during 2010–2012 when their behaviors were self-assessed. Mothers and teachers of these children used the Chinese versions of the youth self report, the child behavior checklist and the teacher report form, respectively, to assess the children’s behaviors. These children reported minor and severe physical abuse experiences in the previous 12 months from mothers and fathers separately using the Chinese version of parent–child conflict tactics scale in 2013 when children were in grades 7 and 8 of middle school.

**Results:**

The present study found that after controlling for the sociodemographic and other cognitive and/or behavior variables, high scores of child externalizing behavior rated by their mothers or teachers were associated with increased risks of experiencing maternal and paternal severe physical abuse, while a high score of self-reported externalizing behavior was associated with a decreased risk of paternal severe physical abuse. A high score of mother-rated internalizing behavior was associated with a decreased risk of maternal severe physical abuse. VIQ was associated with maternal minor physical abuse with small effect size. PIQ was not associated with any forms of physical abuse after adjusting for child behavior and sociodemographic variables.

**Conclusions:**

In this community sample of Chinese children, externalizing behavior perceived by mothers and teachers is linked to children being at risk for physical abuse, while internalizing behavior perceived by mothers is associated with a decreased risk of maternal physical abuse. Findings suggest that educating parents and teachers to appropriately perceive children’s externalizing behavior may help prevent the occurrence of physical abuse.

**Electronic supplementary material:**

The online version of this article (doi:10.1186/s13034-016-0124-5) contains supplementary material, which is available to authorized users.

## Background

Child physical abuse has gained increasing attention in China, especially after the recent enactment of the first national law prohibiting domestic violence (*The Law against Domestic Violence of People’s Republic of China*) in March 2016. Despite being prohibited by the law, child physical abuse is still highly prevalent among Chinese children. A recent meta-analysis of 47 Chinese studies reported that about half of Chinese children have experienced minor physical abuse and about 1 in 5 children have been physically abused [1], which is higher than the estimated global prevalence of physical abuse and the estimated prevalence in Asian countries [2]. Child physical abuse shows associations with increased risks of physical, behavioral, cognitive, and psychological problems during childhood, and such effects can last into adulthood [3, 4]. The adverse consequences related to child abuse, in turn, cause high societal costs in China [3, 4], as they do in other developed countries [5]. However, unlike in developed countries that have launched various prevention programs to prevent child abuse [6, 7], there are very few prevention and intervention programs to protect children against abuse in China. There is a need of research on modifiable risk factors of child abuse to shed lights on developing effective prevention programs in China.

Parent-child interaction is a reciprocal process. While the mainstream research interprets parental abusive behavior as a risk factor for behavioral problems (i.e. a parent effect), it is possible that children with cognitive deficits and behavioral problems may elicit parental abusive behavior (i.e. a child effect). The latter line of explanation is supported by the limited evidence from both cross-sectional and longitudinal studies that found bidirectional relationships between child abuse and behavioral outcomes: on one hand, abused children had more behavioral problems in later childhood after controlling for previous behavioral problems, and, on the other hand, children with behavioral problems were more likely to experience coercive parenting or child abuse after controlling for the previous abuse experiences [8–11]. Similarly, a meta-analysis study also revealed that the “parent perceives child as problem” viewpoint was a risk factor for child physical abuse [12]. In addition, researchers have also found a significant child effect in terms of intelligence. Children with low intelligence quotient (IQ) were at high risk of childhood abuse or exposure to trauma [13–15].

The child effect that child behavior problems elicit parental practice of abuse may be particularly salient in China due to traditional Chinese culture. Chinese culture regards harsh child discipline as necessary to increase children’s morality and obedience to social harmony when they misbehave [16–18]. Leung et al. conducted a large-scale study in southern China and found the most common reason for abuse was “disobedience to parents,” which is usually regarded as misbehavior by Chinese parents [19]. Consistently, a qualitative study found that Chinese parents hold the view that they only practice physical discipline when their children misbehave, and the purpose of the physical discipline is to correct child’s behavior for the child’s good [18]. Even the survivors of child abuse agreed that they were physically abused because they did something wrong [20]. However, the cognitive and behavioral risk factors for child abuse in China have been understudied.

In addition, the present literature is limited because the researchers collected child behavior data from only one informant source, usually either mothers or children, which may not comprehensively capture the complexity of child behavior. Research shows that there is a situational effect of child behavior: parents and teachers may hold different perceptions of child behavior, which is also different from the child’s own perception of his/her behavior [21]. However, it remains unknown whether child behavior perceived by different informants is associated with child physical abuse in a different or similar fashion.

Another limitation in the literature is that most studies assess child abuse as practiced by both of the parents, or only the mother, yet it fails to distinguish child abuse as practiced separately by both the mother and the father. Studies have found gender differences in parenting styles, with mothers demonstrating more authoritative (i.e., emotionally supportive and responsive) parenting styles and fathers exhibiting more authoritarian (i.e., less supportive and high-controlling) parenting styles [22, 23]. In addition, researchers have reported that maternal and paternal parenting has different effects on children’s behavior in China [24, 25]. Therefore, it is necessary to consider maternal and paternal abusive behaviors simultaneously, yet separately.

Therefore, this study aims to examine the associations of previously measured IQ and behavioral problems (reported by mothers, teachers, and children) with later child physical abuse perpetrated separately by mothers and fathers.

## Methods

### Procedures and participants

The present study used secondary data collected from the Wave II of the Jintan Child Cohort Study, which is an ongoing prospective longitudinal study. The cohort study recruited 1385 children aged 3–5 years old from upper grade (i.e. mean age about 5 years old), middle grade (i.e. mean age about 4 years old), and bottom grade (i.e. mean age about 3 years old) in preschools in Jintan, China in 2004–2005, which was a representative sample of children in the city in terms of gender, age, and residential locations. The cohort study design was described elsewhere [26–28].

**Fig. 1:**
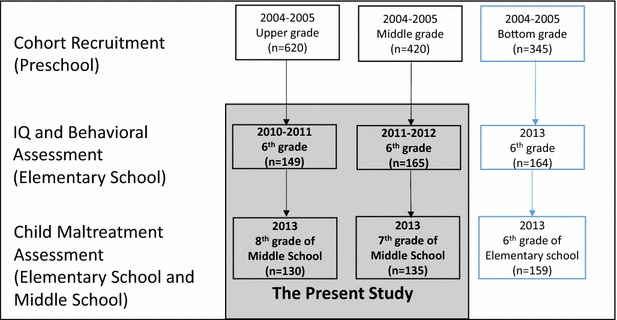
The flow chart of the temporal design of the China Jintan Child Cohort study and the present study. The *gray area* illustrates when the participants’ IQ, behaviors, and child abuse experience were assessed in the present study. The number in each *rectangle* indicates the sample size with complete data on the variables of interest

The children from upper grade, middle grade, and bottom grade were followed up with during the Wave II to assess behavioral problems (reported by children, mothers, and teachers) and IQ in 2010–2011, 2011–2012, and 2013, respectively. All of the children were also invited to participate in a child abuse questionnaire survey in 2013 when children were 6th, 7th and 8th graders. In order to maintain temporal order to test the association of IQ and behavioral problems in earlier life and later child abuse, we included the 7th and 8th graders whose behavioral problems and IQ were assessed in 2010–2011 and 2010–2012, and child physical abuse was assessed in 2013. We obtained complete data from 265 children (47.2 % boys). The temporal design of the parent cohort study and the present study is shown in Fig. 1. Compared with those who did not have complete data, these children did not show significant differences in age, verbal IQ (VIQ), performance IQ (PIQ), or externalizing and internalizing behaviors (regardless of the reporters), or minor or severe physical abuse (regardless of the perpetrators). There were slightly more girls, more children from better socioeconomic background, and fewer children from the rural areas in the retained sample (Additional file 1: Table S1).

We obtained written informed consent from both mothers and teachers and verbal consent from children during the Wave II of data collection. Two trained research assistants distributed and collected the questionnaires, explained the objectives and confidentiality of the study and the principle of voluntary participation and participations’ right of withdrawing the study at any time point, and answered any of the respondents’ questions. All questionnaire surveys for the children took place in classrooms during school hours. Children completed the IQ test at Jintan Hospital and, in the meantime, parents rated their children’s behavior in the waiting rooms. Teachers rated child behavior in their offices after understanding the study. We obtained approval from the Institutional Review Board of the University of Pennsylvania and the Ethical Committee for Research at Jintan Hospital, China.

### Measures

#### Child physical abuse

Children's physical abuse experiences were assessed by the Pa rent–Child Conflict Tactics Scale-child version (CTSPC) [29] in 2013, which consists of 27 items covering four categories of parental behaviors: (1) nonviolent disciplinary behaviors (4 items), (2) psychological aggression (5 items), (3) physical abuse, including minor form (6 items, including spanking with bare hand, hitting bottom with objects, slapping on hand or arm or leg, slapping on face or head or ears, pinching, shaking or pushing) and severe form (7 items, including hitting other part of body besides bottom with objects, throwing or knocking down, hitting with a fist or kicking hard, beating up, choking, burning, threatening with a weapon), and (4) neglect (5 items). Children were asked to provide information on whether their mothers and fathers separately displayed these behaviors in the preceding year (0 = “No”, or 1 = “Yes”). For the purpose of the study, we focused on the minor and severe forms of child physical abuse. Non-abused children were those with zeros on all items in the corresponding subscales. Otherwise, they were labeled as minor or severe physical abuse survivors. The available Chinese version of the CTSPC showed satisfactory to good reliability (0.58–0.87 [30]). The subscales of minor and severe physical abuse showed good reliability for maternal vs. paternal behaviors (minor physical abuse: 0.73 vs. 0.77; physical abuse: 0.69 vs. 0.65) in the study.

#### Child externalizing and internalizing behavior

Three questionnaires from the Achenbach System of Empirically Based Assessment (ASEBA [31, 32]) were used to assess child behavior. Parents and teachers completed the validated Chinese versions of the child behavior checklist for ages 6–18 (CBCL) and the teacher report form (TRF), respectively. Children self-reported their behaviors using the validated Chinese version of Youth Self-Report (YSR). The CBCL and TRF consist of 115 items each, while the YSR consists of 112 items. The questionnaire items were rated on a 3-point scale (0 = not true, 1 = sometimes true, and 2 = often true), from which normalized *T* scores (the ratio of behavior score’s deviation from the population mean to its standard deviation) were calculated. A higher *T* score indicates more behavioral problems. The researchers classified all items into three factors: externalizing behavior, internalizing behavior, and other problems. In the present study, the factors of externalizing behavior (score range in the study: 37.13–87.74) and internalizing behavior (score range in the study: 35.28–110.50) were used in analyses.

#### Cognition

The researchers assessed children’s cognition using the Chinese version of the Wechsler intelligence scale for children-revised (WISC-R), which measured children’s PIQ and VIQ and showed good reliability and validity among Chinese children ages 6–17 years old [33]. Details of the test were described elsewhere [34, 35].

#### Sociodemographic co-variables

Children completed a self-administered General Information Questionnaire to provide information about their gender, age when child abuse was assessed, grade when their abuse experience was assessed, fathers’ and mothers’ number of years of education, and fathers’ and mothers’ monthly wage. Their mothers were asked the current family location (i.e. urban, suburban, or rural) when the children were recruited in the cohort study. We generated an indicator of socioeconomic status (SES) according to the procedure described in [36]. It is the standardized *z* score of the sum of z scores of children’s father’s and mothers’ number of years of education and monthly wage.

### Data analysis

We first ran descriptive analyses for all variables. We described the prevalence of child physical abuse by mothers and fathers, respectively, and compared the intelligence and behavioral characteristics of children with a specific type of abuse to those without it. We then ran variance inflated factor (VIF) analysis to determine the multicollinearity of the independent variables. The result showed that VIF of the six behavior variables ranged from 2.07 to 2.32. Therefore, multicollinearity of behavioral variables was not a severe concern. Age and grade were highly correlated and, thus, only grade was controlled in multivariate analyses. In order to illustrate meaningful odds ratios, we rescaled VIQ, PIQ, and behavior variables by dividing each of them by 10. Therefore, the OR and 95 % confidence intervals indicate a change in the risk of being maltreated with a 10-point increase in VIQ, PIQ, or behavior scores. Using the rescaled IQ and behavior scores as independent variables, we constructed four generalized linear models with binomial family and logit link function to test the association of IQ and behavioral problems with the risk of child physical abuse 1 or 2 years later, controlling for the co-variables. Minor or severe physical abuse by mothers and fathers were treated as dependent variables in the four models, respectively. Next, we constructed GLMs with binomial family and log link to obtain the risk ratio (RR) for the significant cognitive and behavioral factors associated with physical abuse to estimate the effect sizes of their associations with physical abuse. In order to get convergent GLMs with log link, one case with the highest predicted value obtained from the GLMS with logit link was removed for each GLM model with log link. We set the significance level at α = 0.05/4 = 0.125 using the Bonferroni correction of four outcomes, and regarded a p value less than 0.05 but higher than 0.0125 as marginally significant or a trend of significance. We performed all the analyses using STATA 13.0 for Windows (College Station, TX).

## Results

### Sample characteristics

Among the 265 children, almost half of them experienced minor physical abuse by either their mothers or fathers, and about one-fourth of children experienced severe physical abuse from either their mothers or fathers. Boys were more likely to report physical abuse from their fathers than girls (*χ* ^2^ = 6.944, p = 0.008). There is no significant difference between physically maltreated children and their non-maltreated counterparts in terms of age, location, and socioeconomic status. See Table 1.

**Table 1:** Sample characteristics and their association with prevalence of child abuse (*n* = 265)

	Total sample	Maternal minor physical abuse			Paternal minor physical abuse			Maternal severe physical abuse			Paternal severe physical abuse		
	M ± SD/n (%)	Yes	No	*t/χ* ^2^	Yes	No	*t/χ* ^2^	Yes	No	*t/χ* ^2^	Yes	No	*t/χ* ^2^
		131 (49.4)	134 (51.6)		113 (43.1)	149 (56.9)		66 (24.9)	199 (75.1)		63 (23.8)	202 (76.2)	
Gender													
Girls	140 (52.8)	67 (47.8)	73 (52.1)	0.295	55 (40.0)	83 (60.0)	1.275	35 (25.0)	105 (75.0)	0.001	25 (17.8)	115 (82.1)	5.733^†^
Boys	125 (47.2)	64 (51.2)	61 (48.8)		58 (46.8)	66 (53.2)		31 (24.8)	94 (75.2)		38 (30.4)	87 (69.6)	
Grade													
7th	130 (49.1)	68 (50.7)	62 (47.3)	0.310	81 (54.4)	46 (40.7)	4.797^†^	94 (47.2)	36 (54.5)	1.059	96 (47.5)	34 (54.0)	0.798
8th		66 (49.3)	69 (52.7)		68 (45.6)	67 (59.3)		105 (52.8)	30 (45.5)		106 (52.5)	29 (46.0)	
Location													
Urban	121 (45.7)	60 (49.6)	61 (50.4)	0.009	50 (42.0)	69 (58.0)	0.131	29 (24.0)	92 (76.0)	0.114	29 (24.0)	92 (76.0)	0.096
Suburban	116 (43.7)	57 (49.1)	59 (50.9)		51 (44.3)	64 (55.7)		30 (25.9)	86 (74.1)		28 (24.1)	88 (75.9)	
Rural	28 (10.6)	14 (50.0)	14 (50.0)		11 (42.9)	16 (57.1)		7 (25.0)	21 (75.0)		6 (21.4)	22 (78.6)	
Age	13.71 ± 0.60	13.73 ± 0.61	13.70 ± 0.59	0.285	13.77 ± 0.61	13.68 ± 0.59	1.111	13.66 ± 0.64	13.73 ± 0.59	0.866	13.63 ± 0.61	13.74 ± 0.60	1.241
SES	0.06 ± 1.18	0.03 ± 1.19	0.10 ± 1.18	0.457	0.10 ± 1.20	0.04 ± 1.18	0.388	−0.07 ± 1.15	0.11 ± 1.19	1.097	−0.07 ± 1.08	0.11 ± 1.21	1.022

The values displayed in the cells indicate mean ± standard deviations for continuous variables and frequency (percentage) for categorical variables

*SES* socioeconomic status

^†^p < 0.05

### Bivariate associations of child physical abuse with IQ and behavioral problems

Children who experienced maternal minor physical abuse in the preceding year had higher scores of externalizing behaviors as rated by their respective mothers (51.31 ± 9.36 vs. 48.35 ± 7.97, *p* = 0.006, Cohen’s *d* = 0.34) and themselves (50.92 ± 10.25 vs. 47.85 ± 8.76, *p* = 0.009, Cohen’s *d* = 0.32). Children with paternal minor (51.69 ± 9.52 vs. 48.50 ± 8.00, *p* = 0.003, Cohen’s *d* = 0.37) or severe physical abuse (52.32 ± 9.69 vs. 49.03 ± 8.37, *p* = 0.009, Cohen’s *d* = 0.38) scored higher on externalizing behaviors as rated by their mothers in the past. The effect sizes of these differences are small to medium. Children with an experience of maternal severe physical abuse showed a trend of lower PIQ scores, higher externalizing behaviors scores rated by their teachers, and higher self-reported internalizing behavior scores, while children with paternal severe physical abuse showed a trend of higher scores on teacher-rated externalizing behavior (Table 2). However, these results did not reach the significance level at 0.0125.

**Table 2:** Abused children’s IQ and behavior problems (*n* = 265)

	PIQ	VIQ	S_EXTER	M_EXTER	T_EXTER	S_INTER	M_INTER	T_INTER
Maternal minor physical abuse								
Yes	105.30 ± 11.64	100.77 ± 11.48	50.92 ± 10.25	51.31 ± 9.36	50.23 ± 8.74	49.99 ± 10.60	51.20 ± 10.89	50.54 ± 10.44
No	106.71 ± 12.62	101.30 ± 11.40	47.85 ± 8.76	48.35 ± 7.97	49.56 ± 7.84	47.58 ± 9.31	48.62 ± 8.80	50.64 ± 10.16
Cohen’s d	0.12	0.05	0.32*	0.34*	0.08	0.24^†^	0.26^†^	0.01
Paternal minor physical abuse								
Yes	105.327 ± 11.83	100.29 ± 11.44	49.31 ± 10.09	51.69 ± 9.52	49.61 ± 8.54	48.73 ± 9.94	51.65 ± 9.00	49.77 ± 9.62
No	106.23 ± 12.11	101.36 ± 11.33	49.49 ± 9.37	48.50 ± 8.00	50.19 ± 8.16	48.92 ± 10.17	48.72 ± 10.93	51.30 ± 10.80
Cohen’s d	0.08	0.09	0.02	0.37*	0.07	0.02	0.30^†^	0.15
Maternal severe physical abuse								
Yes	103.24 ± 12.52	99.74 ± 11.60	50.96 ± 9.82	51.35 ± 8.75	51.85 ± 10.20	50.96 ± 11.59	50.59 ± 9.70	52.50 ± 9.25
No	106.93 ± 11.91	101.47 ± 11.37	48.84 ± 9.53	49.31 ± 8.77	49.25 ± 7.50	48.05 ± 9.36	49.66 ± 10.05	49.96 ± 12.78
Cohen’s d	0.31^†^	0.15	0.22	0.23	0.32^†^	0.29^†^	0.09	0.25
Paternal severe physical abuse								
Yes	106.19 ± 12.09	101.05 ± 11.73	49.21 ± 9.03	52.32 ± 9.69	52.16 ± 10.40	48.82 ± 9.92	51.63 ± 10.63	52.23 ± 12.02
No	105.96 ± 12.19	101.03 ± 11.35	49.42 ± 9.83	49.03 ± 8.37	49.19 ± 7.41	48.76 ± 10.07	49.35 ± 9.69	50.08 ± 9.65
Cohen’s d	0.02	0.001	0.02	0.38^*^	0.36^†^	0.007	0.23	0.21

*PIQ* performance intelligence quotient; *VIQ* verbal intelligence quotient; *S_EXTER* child self-report externalizing behavior; *M_EXTER* mother-report externalizing behavior; *T_EXTER* teacher-report externalizing behavior; *S_INTER* child self-report internalizing behavior; *M_INTER* mother-report internalizing behavior; *T_INTER* teacher-report internalizing behavior

^†^ *p* < 0.05; * *p* < 0.0125

### The adjusted association of IQ and behavioral problems with later physical abuse

Table 3 illustrates the adjusted associations of child physical abuse with IQ and behavior problems. After adjusting for other variables in the model, the risk of maternal severe physical abuse increased with the increase in the scores of mother—[OR = 1.38 (1.09, 1.74), *p* = 0.007, RR = 1.28] or teacher—[OR = 1.47 (1.29, 1.69), *p* = 0.009, RR = 1.22] rated externalizing behavior, while such risk decreased with the increase in the score of mother-rated internalizing behavior [OR = 0.77 (0.63, 0.95), *p* = 0.011, RR = 0.79]. Similarly, the risk of paternal severe physical abuse grew with the increase in the scores of mother—[OR = 1.47 (1.29, 1.69), *p* < 0.001, RR = 1.31] or teacher-rated externalizing behavior [OR = 1.61 (1.44–1.81), *p* < 0.001, RR = 1.32]. Although a higher score of VIQ was related to increased risk of maternal minor physical abuse [OR = 1.06 (1.02–1.13), *p* = 0.006, RR = 0.04], the effect size was very small. Notably, the ORs change with the increase in behavior scores. For example, with an increase of 20 points in mother-rated externalizing behavior, the odds of maternal severe physical abuse increases from 1.38 to 1.90 (RR increases from 1.28 to 1.64), compared to the odds of not experiencing such abuse. Neither IQ nor behavioral problems rated by different informants were significantly associated with the risk of paternal minor physical abuse.

**Table 3:** The adjusted associations of IQ and behavior problems with physical abuse (*n* = 265)

	Maternal minor physical abuse	Paternal minor physical abuse	Maternal severe physical abuse	Paternal severe physical abuse
	Adjusted OR (95 % CI)	Adjusted OR (95 % CI)	Adjusted OR (95 % CI)	Adjusted OR (95 % CI)
VIQ	1.07 (1.02, 1.13)*	0.93 (0.76, 1.13)	1.07 (0.61, 1.87)	1.14 (0.99, 1.33)^†^
PIQ	0.87 (0.59, 1.27)	0.88 (0.70, 1.12)	0.78 (0.37, 1.63)	1.03 (0.80, 1.33)
S_EXTER	1.46 (1.05, 2.03)^†^	1.15 (0.93, 1.43)	0.88 (0.54, 1.45)	0.59 (0.52, 0.66)**
M_EXTER	1.33 (0.79, 2.25)	1.36 (0.89, 2.07)	1.38 (1.09, 1.74)*	1.47 (1.29, 1.69)**
T_EXTER	1.11 (0.91, 1.35)	1.03 (0.93, 1.15)	1.45 (1.10, 1.91)*	1.61 (1.44, 1.81)**
S_INTER	1.02 (0.85, 1.22)	0.85 (0.60, 1.21)	1.41 (0.96, 2.06)	1.20 (0.57, 2.51)
M_INTER	1.02 (0.68, 1.75)	1.13 (0.78, 1.64)	0.77 (0.63, 0.95)*	0.97 (0.66, 1.41)
T_INTER	0.81 (0.68, 0.97)^†^	0.86 (0.64, 1.15)	0.88 (0.79, 0.98)^†^	0.94 (0.75, 1.16)

*OR* odds ratio. OR values indicate a 10 point increase in IQ or behavior problems was associated with the change in the likelihood of being physically maltreated. IQ (range: 73–149) and behavioral (range: 35–92) independent variables are treated as continuous variables. *95* *% CI* 95 % confidence interval. Models were adjusted for child gender, age, and socioeconomic status and clustered at location level to correct standard errors

*PIQ* performance intelligence quotient; *VIQ* verbal intelligence quotient; *S_EXTER* child self-report externalizing behavior; *M_EXTER* mother-report externalizing behavior; *T_EXTER* teacher-report externalizing behavior; *S_INTER* child self-report internalizing behavior; *M_INTER* mother-report internalizing behavior; *T_INTER* teacher-report internalizing behavior

^†^ *p* < 0.05; * *p* < 0.0125; ** *p* < 0.001

## Discussion

To our best knowledge, this study is the first to report the association of cognition measured by VIQ, PIQ, and child behavior rated by different informants with maternal and paternal physical abuse in a cohort sample of children. Although the majority of the participated children showed normal intelligence and behavior scores, within these children, we found that children with high scores of mother- and teacher-rated externalizing behavior were more likely to be severe physically abused by their mothers and fathers, while children with high scores on self-rated externalizing behavior were less likely to be severely physically abused by their fathers. Besides, children with high scores of mother-rated internalizing behavior were less likely to report maternal severe physical abuse in later childhood. PIQ was not associated with any form of child physical abuse. It should be noted that the present study does not suggest that children should be blamed for their abuse by their parents. Instead, findings from the study are expected to help better understand risk factors for child abuse, and, therefore, provide evidence for future prevention programs.

### Externalizing behavior and maternal and paternal physical abuse

The present study found that mothers’ and teachers’ reports on externalizing behavior were associated with both maternal and paternal severe physical abuse. This is consistent with the finding from a longitudinal Chinese study that children with high externalizing behavior experienced more physical abuse 6 months later after controlling for the previous physical abuse experience [37]. Similarly, Stith et al. conducted a meta-analysis and reported that child externalizing behavior is a risk factor for child abuse [12]. In terms of the effect size of the association between externalizing behavior and severe physical abuse, the odds ratios are comparable to the estimates from a meta-analysis of 68 Chinese studies treating child abuse as a risk factor for behavioral outcomes. This meta-analysis found that the effect sizes of the associations between child abuse and behavioral outcomes (e.g. mental health disorders, depression, anxiety, drug use, etc.) range from 1.40 to 1.98 [4]. Taken together, the findings indicate that externalizing behaviors perceived by parents or teachers may increase parents’ negative attributions of child behavior that directly increases parenting stress [38] and the tendency of practicing harsh disciplining strategy to correct children’s misbehavior or to reduce their distress.

Interestingly, child self-report externalizing behavior decreased the risk of paternal severe physical abuse. It is possible that Chinese fathers may regard child self-reported externalizing behavior as normal extroversion, and therefore, are less likely to practice severe physical discipline when their mothers’ and teachers’ perceptions of child externalizing behavior are adjusted. Very few studies have attempted to examine the association between child behavior and paternal physical abuse, and more studies are needed.

The findings also suggest that there is a discrepancy in the perceptions of externalizing behavior between children and their parents and teachers. Research found that children usually report fewer behavior problems than their parents or other informants [39]. The disparate perspectives of externalizing behavior may be a source of conflict that triggers parental physical abuse. Hence, it may be effective to prevent child abuse by modifying parents’ and teachers’ perceptions of child behavior.

### Internalizing behavior and maternal severe physical abuse

We found that mother-rated internalizing behavior was associated with less risk of maternal minor or severe physical abuse. Literature from western studies indicates that physically abusive mothers usually rated higher on child internalizing behavior [12, 40], an inconsistent result with the present finding. This inconsistency may indicate that Chinese parents tend not to use physical discipline when they perceive that their children are introverted. Previous research has argued that from the perspective of Chinese parents, the characteristics of internalizing problems may align with desired characteristics in Chinese culture, such as being quiet and sensitive [37, 41]. Therefore, mother-perceived internalizing behavior relates to less-frequent physical abuse.

### IQ and physical abuse

Although the positive association between VIQ and maternal minor physical abuse (that is independent of behavioral problems and sociodemographic variables) was statistically significant, the effect size is very small. We did not find significant associations of VIQ with other types of physical abuse or significant associations of PIQ with all types of physical abuse. The previous findings of the association between IQ and child abuse under the assumption of the child effect are not conclusive. Breslau et al. conducted a longitudinal study and found that full-scale IQ lower than 115 at the age of 6 increased the risk of exposure to general assaultive violence at age 17, and they explained that children with low IQs might be more likely to interact with disruptive peers and, therefore, be exposed to assaultive violence [14]. In contrast, Brown et al. [42] and Young et al. [13] found that low IQ scores were associated with child neglect but not physical abuse, indicating that different types of child abuse may be associated with IQ differently. Further research can be conducted to examine the relationship between IQ and other forms of child abuse other than physical abuse in the Chinese context.

The absence of the significant association between IQ and physical abuse could also be because child behavior fully mediates the relationship between IQ and child physical abuse. Prior studies have suggested that children with intellectual disabilities are at higher risk of developing behavioral problems that may further make children more prone to physical abuse [43, 44]. Future research is warranted to explore the possible mediating role of behavioral problems in the relationship between IQ and child abuse.

### Study limitations

The findings should be interpreted cautiously due to study limitations. First, a relatively small proportion of the original cohort children participated in the survey, and there were slightly more girls and less children from rural areas (Additional file 1: Table S1). Therefore, the present study’s generalizability is limited. Despite this, the present study does exhibit value in offering a new perspective to investigate the relationship between IQ, behavioral problems, and child abuse.

Second, we did not examine gender differences in the relationships of child physical abuse with IQ and behavioral problems concerning low statistical power. Prior studies suggest that there are gender differences in the predictive effect of externalizing and internalizing behavior on physical abuse among Chinese children. Specifically, compared with Chinese girls, Chinese boys with behavioral problems were more likely to experience physical abuse [37, 41]. Future studies are needed to explore whether the association between IQ and child abuse depends on child gender.

Third, we only collected information on child abuse once. The status of child abuse prior to the study was not assessed. It is possible that maltreated children in the present sample had also experienced abuse before the study, and such experience may serve as a confounder in the relationship between behavioral problems and child physical abuse. However, given the findings from the qualitative studies in the Chinese context that Chinese parents practice harsh discipline towards children because of their misbehavior, disobedience, and poor academic performance [18, 20, 45], as well as the bidirectional relationship between child abuse and behavioral problems revealed from the longitudinal studies [8, 9, 11], it is plausible to regard IQ and child externalizing and internalizing behavior as potential risk factors for child abuse. It is worth noting that the majority of studies regarding child abuse as a risk factor for behavioral problems failed to control for previous behavioral problems. Therefore, we suggest that future research further explore the reciprocal relationship of child abuse with cognition and behavior.

Lastly, some confounders that were not included in the present study need to be considered for future studies. For example, parental mental health status could be an important confounder that is related to both child abuse [46] and child behavioral problems [47]. However, very few Chinese researchers have attempted to examine the effect of parental mental health status on child abuse, and, therefore, this needs more attention.

## Conclusions

The study using a community sample of Chinese children found that, even within children with normal intelligence and behavior, relatively more externalizing behavior as rated by teachers and mothers are risk factors for children experiencing physical abuse from both mothers and fathers. In contrast, child internalizing behavior as rated by mothers and teachers may decrease the risk of maternal minor physical abuse due to Chinese beliefs surrounding introversion. IQ is not associated with any forms of physical abuse. The study findings may suggest that it is important to educate teachers and parents to assess and interpret children’s behavior appropriately and to communicate with children about their perceptions of their behavior to prevent parent–child conflicts and, in turn, to prevent child abuse.

## Additional file

10.1186/s13034-016-0124-5 Comparisons of sample characteristics between children that were included (n = 265) and excluded (n = 775) in the study.

## Abbreviations

IQ: intelligence quotient
VIQ: verbal intelligence quotient
PIQ: performance intelligence quotient
CTSPC: parent–child conflict tactics scale
YSR: youth self report
CBCL: child behavior checklist
TRF: teacher report form
WISC-R: Wechsler intelligence scale for children-revised
SES: socioeconomic status
GLM: generalized linear model
OR: odds ratio
RR: risk ratio
CI: confidence interval
S_EXTER: self-report externalizing behavior
M_EXTER: mother-report externalizing behavior
T_EXTER: teacher-report externalizing behavior
S_INTER: self-report internalizing behavior
M_INTER: mother-report internalizing behavior
T_INTER: teacher-report internalizing behavior
