# Supplementary material for: Cognitive and behavioral risk factors for child physical abuse among Chinese children: a multiple-informant study
Source: Child Adolesc Psychiatry Ment Health. 2016 Oct 6;10:36. doi: 10.1186/s13034-016-0124-5 (PMC5052977; doi:10.1186/s13034-016-0124-5)
Supplement: Supplementary file 1 — 10.1186/s13034-016-0124-5 Comparisons of sample characteristics between children that were included (n = 265) and excluded (n = 775) in the study. [file 13034_2016_124_MOESM1_ESM.docx]

**Supplemental Table. Comparisons of sample characteristics between children that were included (n=265) and excluded (n=775) in the study**

|  |  | Included | Excluded | *t/χ^2^* | *p* |
| --- | --- | --- | --- | --- | --- |
| **Sociodemogrphic Characteristics** | | | | | |
| Gender (*n_e_ ^a^*=775) | Girls | 140 (52.8) | 333 (43.0) | 7.75 | 0.005 |
|  | Boys | 125 (47.2) | 442 (57.0) |  |  |
| Grade (*n_e_ ^a^*=775) | 6^th^ grade | 117 (44.2) | 304 (39.23) | 1.99 | 0.159 |
|  | 7^th^ grade | 148 (55.8) | 471 (60.77) |  |  |
| Location (*n_e_ ^a^*=775) | Urban | 121 (45.7) | 278 (35.9) | 34.60 | <0.001 |
|  | Suburban | 116 (43.7) | 277 (35.7) |  |  |
|  | Rural | 28 (10.6) | 220 (28.4) |  |  |
| Age (*n_e_ ^a^*=775) |  | 13.71±0.60 | 13.77±0.91 | 1.40 | 0.161 |
| SES (*n_e_ ^a^*=775) |  | 0.06±1.18 | -0.16±1.00 | 3.01 | 0.003 |
| **Child Physical Abuse** |  |  |  |  |  |
| Maternal minor physical abuse (*n_e_ ^a^*=405) | No | 134 (50.6) | 208 (51.4) | 0.02 | 0.881 |
|  | Yes | 131 (49.4) | 197 (48.6) |  |  |
| Paternal minor physical abuse (*n_e_ ^a^*=405) | No | 149 (56.9) | 225 (55.6) | 0.11 | 0.738 |
|  | Yes | 113 (43.1) | 180 (44.4) |  |  |
| Maternal minor physical abuse (*n_e_ ^a^*=405) | No | 199 (75.1) | 318 (78.5) | 1.24 | 0.266 |
|  | Yes | 66 (24.9) | 87 (21.5) |  |  |
| Paternal minor physical abuse (*n_e_ ^a^*=405) | No | 202 (76.2) | 320 (79.0) | 0.34 | 0.559 |
|  | Yes | 63 (23.8) | 85 (21.0) |  |  |
| **Intelligence** |  |  |  |  |  |
| VIQ (*n_e_ ^a^* =222) |  | 101.04±11.42 | 98.86±13.17 | 1.95 | 0.051 |
| PIQ (*n_e_ ^a^* =222) |  | 106.01±12.14 | 104.65±12.45 | 1.22 | 0.223 |
| **Child Behavior** |  |  |  |  |  |
| S_EXTER (*n_e_ ^a^* =269) |  | 49.37±9.63 | 48.90±9.82 | 0.56 | 0.57 |
| M_EXTER (*n_e_ ^a^* =211) |  | 49.82±8.79 | 49.61±9.93 | 0.24 | 0.809 |
| T_EXTER (*n_e_ ^a^* =246) |  | 49.89±8.29 | 50.77±49.45 | 1.05 | 0.292 |
| S_INTER (*n_e_ ^a^* =269) |  | 48.77±10.02 | 49.55±9.29 | 0.93 | 0.353 |
| M_INTER (*n_e_ ^a^* =211) |  | 49.89±9.95 | 50.16±9.64 | 0.29 | 0.768 |
| T_INTER (*n_e_ ^a^* =246) |  | 50.59±10.28 | 50.65±9.91 | 0.07 | 0.94 |

*Notes. a, n_e_* represents the sample size of excluded children with relevant data*.* The values displayed in the cells indicate mean ± standard deviations for continuous variables and frequency (percentage) for categorical variables. SES, socioeconomic status. PIQ: performance intelligence quotient; VIQ: verbal intelligence quotient; S_EXTER, child self-report externalizing behavior; M_EXTER, mother-report externalizing behavior; T_EXTER, teacher-report externalizing behavior; S_INTER, child self-report internalizing behavior; M_INTER, mother-report internalizing behavior; T_INTER, teacher-report internalizing behavior.
